# Expression of concern: Effects of microRNA-374 on proliferation, migration, invasion and apoptosis of human SCC cells by targeting Gadd45a through P53 signaling pathway

**DOI:** 10.1042/BSR-20170710_EOC

**Published:** 2020-08-25

**Keywords:** Apoptosis, Gadd45a, MicroRNA-374, P53 signaling pathway, Proliferation, Squamous carcinoma cells

The Editorial Office has been made aware of potential issues surrounding the scientific validity of this paper, hence has issued an expression of concern to notify readers whilst the Editorial Office investigates.

